# Influence of ^60^Co Irradiation on the Volatile Organic Compounds of Cnidii Fructus

**DOI:** 10.3390/metabo16050309

**Published:** 2026-04-30

**Authors:** Junmei Huang, Yuhuan Liu, Yuqing Liu, Jianye Yan, Shunxiang Li, Dan Huang

**Affiliations:** 1 State Key Laboratory of Chinese Medicine Powder and Medicine Innovation in Hunan (Incubation), Academy of Chinese Medical Sciences, Hunan University of Chinese Medicine, Changsha 410208, China; huangjunmei@stu.hnucm.edu.cn (J.H.); yuhuan-liu@stu.hnucm.edu.cn (Y.L.); liuyuqing@stu.hnucm.edu.cn (Y.L.); yanjy@hnucm.edu.cn (J.Y.); 2 School of Pharmacy, Hunan University of Chinese Medicine, Changsha 410208, China; 3 Hunan Province Sino-US International Joint Research Center for Therapeutic Drugs of Senile Degenerative Diseases, Hunan University of Chinese Medicine, Changsha 410208, China; 4 Hunan Engineering Technology Research Center for Bioactive Substance Discovery of Chinese Medicine, Changsha 410208, China

**Keywords:** Cnidii Fructus, Gas Chromatography–Ion Mobility Spectrometry, ^60^Co, irradiation sterilization, volatile organic compounds, multivariate statistical analysis

## Abstract

Background/Objectives: As a traditional Chinese medicinal herb, Cnidii Fructus is widely used in clinical practice. Its volatile organic compounds (VOCs) are closely related to its antipruritic effect and insecticidal properties. Due to the susceptibility of this medicinal herb to mold contamination, adopting appropriate sterilization measures is of great significance for its storage. ^60^Co irradiation is widely used for this purpose due to its various advantages. Methods: This study employed Gas Chromatography–Ion Mobility Spectrometry (GC-IMS) combined with multivariate statistical analysis to systematically investigate the influence of different ^60^Co irradiation doses (0, 3, 6, 9 kGy) on the VOCs of Cnidii Fructus and associated metabolic regulatory mechanisms. Results: A total of 115 VOCs were tentatively identified. Statistical analysis revealed dose-dependent effects: 3 kGy irradiation caused the least compositional perturbation, best preserving original chemical characteristics; 6 kGy induced more pronounced compositional changes; and 9 kGy triggered substantial chemical composition reconstruction. Differential metabolite enrichment analysis indicated that medium and high doses of irradiation primarily perturbed central carbon metabolic pathways, including pyruvate metabolism, glycolysis/gluconeogenesis, and glyoxylate and dicarboxylate metabolism. Key differential components were tentatively identified (e.g., α-Thujone, α-Pinene, β-Pinene) that possess pharmacological activities closely associated with the traditional efficacy of Cnidii Fructus. Conclusions: When the irradiation dose is 3 kGy, the VOCs profile of Cnidii Fructus is most similar to that of the non-irradiated control group, suggesting that its compositional profile may be closer to that of traditional high-quality medicinal materials. Meanwhile, the differential metabolites and core metabolic pathways identified in this study can provide a chemical reference for the quality control of irradiated Cnidii Fructus. The findings provide a theoretical basis and technical support for the rational application of ^60^Co irradiation sterilization in the processing of Chinese medicinal materials and their powders.

## 1. Introduction

Cnidii Fructus, commonly used in Asian countries such as China, Korea, Japan, and Vietnam, is derived from the dried ripe fruit of *Cnidium monnieri* (L.) Cuss [1] and has traditionally been used to treat skin conditions such as atopic dermatitis [2,3].

More than 400 chemical constituents have been identified in Cnidii Fructus. These include coumarins, flavonoids, and volatile oils [4,5]. Recent pharmacological research has highlighted the diverse bioactivities of Cnidii Fructus, including anti-inflammatory [2], anti-tumor [6], sedative and hypnotic [7], anti-osteoporotic [1], and insecticidal and anti-pruritic effects [4,8]. The insecticidal and anti-itching effects of Cnidii Fructus have proven to be remarkably effective in the treatment of atopic dermatitis and have demonstrated broad application prospects in the therapeutic management of various dermatological conditions, including contact dermatitis, tinea corporis and cruris, and pruritus ani [8,9,10]. The volatile organic compounds (VOCs) of volatile oil constitute one of the primary active constituents through which Cnidii Fructus exerts these effects.

As a traditional Chinese medicinal material, Cnidii Fructus is primarily administered externally in clinical practice. It is often used in its raw form or as a raw powder to exert its insecticidal and anti-pruritic effects. However, due to its high content of carbohydrates and other constituents, it is highly susceptible to mold contamination and bacterial proliferation during storage, especially when exposed to unfavorable environmental temperatures and humidity. This not only degrades the active pharmaceutical ingredients (such as osthole) but may also lead to the production of hazardous metabolites like aflatoxins, posing a serious threat to clinical medication safety [11]. Therefore, it is of great significance to explore the processing technology of Cnidii Fructus.

Sterilization is a critical process in food and pharmaceutical manufacturing, necessary to eliminate harmful microbial contamination and ensure product safety, stability, and extended shelf life. Traditional sterilization approaches for traditional Chinese medicine powders have included both thermal sterilization [12,13] and non-thermal sterilization methods [14,15,16]. Of these methods, moist heat sterilization uses high-temperature steam conduction and may incompletely sterilize the interior due to powder clumping. Dry heat sterilization requires temperatures of 160–180 °C, which can cause powder charring or active ingredient degradation. Residual ethylene oxide from gas fumigation may cause allergic reactions and necessitates a closed working environment and prolonged operation times. Ethanol maceration can cause the loss of water-soluble components and is suitable only for small-scale laboratory operations. In recent years, ^60^Co radiation sterilization technology has gained popularity due to its strong penetrating power, applicability at room temperature, high sterilization efficiency and uniformity, absence of harmful residues, and ability to preserve traditional Chinese medicine efficacy [16]. This technology offers a green, efficient, and safe solution for sterilizing traditional Chinese medicine powders. Common radiation doses include 3 kGy and 6 kGy. Moreover, studies have shown that 3 kGy is sufficient for effective sterilization, inactivating microorganisms without risk of reactivation [17,18,19]. Therefore, this study will explore the effects of radiation doses of 3 kGy, 6 kGy, and 9 kGy on the VOCs of Cnidii Fructus.

Common analytical methods for VOCs currently include gas chromatography–mass spectrometry (GC-MS) [20], electronic nose (E-Nose) [21], and Gas Chromatography–Ion Mobility Spectrometry (GC-IMS) [22]. Among these, GC-IMS has technical advantages over traditional methods due to its rapid detection response, trace-level sensitivity, user-friendly interface, and significantly reduced operational costs [23]. Compared to conventional GC-MS, GC-IMS offers higher sensitivity and resolution, faster analysis speed, and simpler operation and eliminates the need for high vacuum conditions, making it particularly suitable for non-targeted rapid screening of VOCs [24].

Although ^60^Co irradiation has been utilized for sterilizing herbal materials [25,26,27], previous research has primarily focused on non-volatile marker compounds and microbial safety, largely disregarding the impact on VOCs. This oversight is especially critical for Cnidii Fructus, where VOCs comprise the essential oil fraction and play a pivotal role in its therapeutic efficacy against skin disorders. Therefore, understanding the alterations in VOCs induced by irradiation is essential for assessing the quality and clinical suitability of this medicinal material. While prior studies, such as Xiang et al. (2023) [27], have explored the effects of irradiation on the VOC profiles of other medicinal plants (e.g., Citri sarcodactylis fructus) and documented significant compositional changes at high doses (10 kGy), critical limitations persist. These investigations failed to elucidate the underlying metabolic pathways of VOC alterations or their pharmacological implications. To address these gaps, this study innovatively investigates the dose-dependent effects of ^60^Co irradiation (0, 3, 6, and 9 kGy) on the comprehensive VOC profile of Cnidii Fructus using GC-IMS combined with multivariate statistical analysis. By deciphering the associated metabolic patterns, this work deepens the understanding of irradiation’s impact on the quality and efficacy of the medicinal material and provides a theoretical foundation for the rational application of irradiation preservation technology in traditional Chinese medicine.

## 2. Materials and Methods

### 2.1. Materials

Cnidii Fructus was collected from Taian, Shandong, China, and it was identified by Prof. Zhaoming Xie from the Hunan Academy of Traditional Chinese Medicine. A voucher specimen (HNATCM2025-SCZ001) was deposited in the herbarium of the same institution.

### 2.2. ^60^Co Radiation

The samples were subjected to total irradiation doses of 0, 3, 6, and 9 kGy. The ^60^Co radiation source was located at the Hunan Radiological Technology Application Research Center (Changsha, China). Sample 1 was exposed to 0 kGy and named SCZ-01, sample 2 was exposed to 3 kGy and named SCZ-02, sample 3 was exposed to 6 kGy and named SCZ-03, and sample 4 was exposed to 9 kGy and named SCZ-04.

### 2.3. Analysis by GC–IMS

Instruments and equipment: FlavourSpec^®^ Gas-Phase Ion Mobility Spectrometer, G.A.S. (Dortmund, Germany); CTC-PAL 3 Static Headspace Autosampler, CTC Analytics AG (Zwingen, Basel, Switzerland); VOCal data processing software (version 0.4.10), G.A.S. (Dortmund, Germany). Six ketones (2-butanone, 2-pentanone, 2-hexanone, 2-heptanone, 2-octanone, and 2-nonanone, each is 10 ppm, Analytical Reagent) were detected, and a calibration curve of retention time and retention index was established.

#### 2.3.1. Sample Preparation

The volatile oil was extracted via steam distillation, referring to the Chinese Pharmacopoeia 2025 edition (part 4) [28]. We added 300 g of dried Cnidii Fructus with an appropriate amount of water and a few glass beads into a 3000 mL round-bottom flask. The mixture was slowly heated to boiling and then kept at a slight boil for 6 h. The upper oil phase (the crude volatile oil of Cnidii Fructus) was collected and sealed, then stored at 4 °C away from light for further use.

For each sample, 10 µL of volatile oil was transferred into a 20 mL headspace vial and incubated at 80 °C for 20 min prior to injection. Three independent biological replicates were included in the experiment.

#### 2.3.2. Headspace Conditions

Incubation temperature: 80 °C; Incubation time: 20 min; Injection volume: 100 µL; Splitless injection; Incubation speed: 500 r/min; Syringe temperature: 85 °C.

#### 2.3.3. GC Conditions

MXT-WAX capillary column (30 m × 0.53 mm, 1.0 μm), Restek Corporation, Bellefonte, PA, USA. Column temperature: 60 °C; Carrier gas: high-purity nitrogen (purity ≥ 99.999%); Programmed pressure: initial flow rate of 2.0 mL/min held for 2 min, linearly increased to 10.0 mL/min over 8 min, then linearly increased to 100.0 mL/min over 10 min, and held for 40 min. Total chromatographic run time: 60 min; Inlet temperature: 80 °C. The drift gas flow rate was maintained constant at 75.0 mL/min.

#### 2.3.4. IMS Conditions

Ionization source: Tritium (^3^H); Drift tube length: 53 mm; Electric field strength: 500 V/cm; Drift tube temperature: 45 °C; Drift gas: high-purity nitrogen (purity ≥ 99.999%); Flow rate: 75.0 mL/min; Positive ion mode.

### 2.4. Statistical Analysis

Analysis of VOCs was performed using VOCal data processing software (version 0.4.10, G.A.S., Dortmund, Germany). This software incorporates multiple plugins enabling the generation of three-dimensional spectra, two-dimensional top views, difference spectra, and fingerprint plots. Partial least squares discriminant analysis (PLS-DA) was conducted with SIMCA software (version 14.1, Umetrics, Malmö, Sweden). Compounds exhibiting significant differences were identified using OriginPro software (version 10.1.5.132, OriginLab Corporation, Northampton, MA, USA). Metabolite enrichment analysis was performed via the MetaboAnalyst platform (https://www.metaboanalyst.ca (accessed on 9 March 2026)). The significance of differences and fold changes was calculated using Microsoft Excel. The volcano plot was generated using the MicroLife Science online platform (https://bioinformatics.com.cn (accessed on 9 March 2026)).

## 3. Results

### 3.1. Analysis of GC-IMS

#### 3.1.1. Comparative Analysis of VOCs in Cnidii Fructus Subjected to Varying Intensities of ^60^Co Irradiation

**Figure 1:**
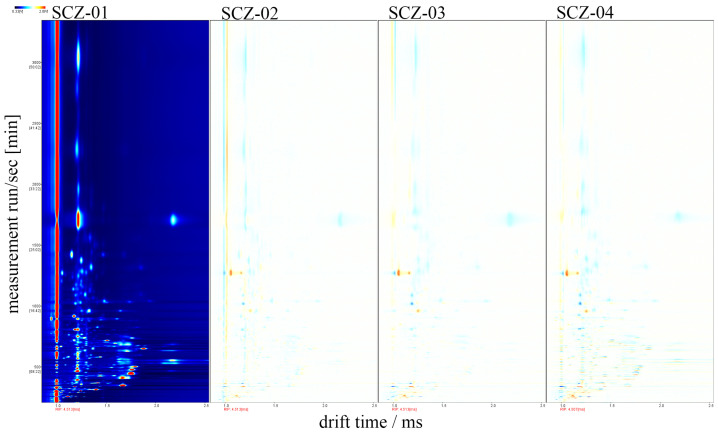
Difference spectra of VOCs by GC-IMS.

To facilitate a more intuitive comparison of the effects of varying intensities of ^60^Co irradiation on the VOCs in Cnidii Fructus, the spectrum obtained from the unirradiated Cnidii Fructus sample (SCZ-01) was used as a reference. The spectra of the other samples were subtracted from this reference spectrum to generate difference comparison plots (Figure 1). In these plots, a white background indicates that the VOC content in the target sample is the same as in the reference sample. Red regions signify a higher concentration of a particular substance in the target sample compared to the reference, whereas blue regions indicate a lower concentration. These difference spectra further reveal distinct variations in the volatile components among Cnidii Fructus samples treated with different intensities of ^60^Co irradiation.

#### 3.1.2. Qualitative Analysis of VOCs in Cnidii Fructus Subjected to Different Intensities of ^60^Co Irradiation by GC-IMS

This study tentatively identified a total of 115 VOCs, including 24 ketones, 20 alcohols, 18 esters, 11 aldehydes, 10 monoterpenes, 10 alkenals, 8 pyrazines, 6 aromatic hydrocarbons, 3 sulfur compounds, 3 furans, 1 acid, and 1 enol; the details are shown in Table 1.

#### 3.1.3. Fingerprint Analysis of VOCs in Cnidii Fructus with Different ^60^Co Irradiation Intensities

**Figure 2:**
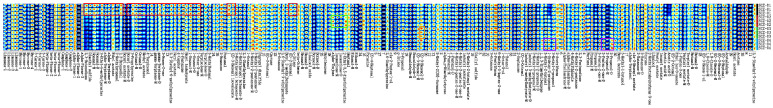
Fingerprint spectrum of VOCs.

To compare differences in volatile substances among Cnidii Fructus samples treated with varying intensities of ^60^Co irradiation, fingerprint analysis was performed on all VOCs; the results are presented in Figure 2. Each row in the figure corresponds to all signal peaks for a given sample, while each column represents the signal peak of a specific VOC across different samples. Brighter colors indicate higher content of the compound. As shown in Figure 2, the differences in volatile components among the four sample groups are clearly visible: The SCZ-01 sample was characterized by abundant levels of compounds such as 2,3-Butanedione, Dimethyl sulfide, Nonanal-M, Nonanal-D, 2-Ethyl-5-methylpyrazine, α-Terpinene-M, α-Terpinene-D, Ethyl acetate, 2-Methylpropanal, 2,3-Butanediol, Bornyl acetate-D, 2-Propylpyrazine, Octanal-M, Octanal-D, Methyl acetate, 4-Terpinenol, γ-Terpinene-M, γ-Terpinene-D, 3-Nonen-2-one, 2,3-Diethyl-6-methylpyrazine, Neryl acetate, d-Camphor, 2-Pentanone, Hexyl isobutyrate, 1-Hexanol-M, 1-Hexanol-D, 1-Octen-3-one, (Z)-3-Hexenyl isovalerate, (E)-2-Nonenal, and o-Xylene. The SCZ-02 sample was distinguished by higher levels of α-Thujone, Isomenthone-M, Isomenthone-D, and 2-Ethyl-3,5-dimethylpyrazine. The SCZ-03 sample exhibited relatively higher levels of compounds such as (Z)-3-Hexenol-D. The SCZ-04 sample was characterized by higher contents of 3-Methyl-2-butenal, 1-Hydroxy-2-propanone, 2-Methyl-1-propanol, 3-Penten-2-one, 2-Propanol-M, and 2-Propanol-D.

### 3.2. Multivariate Statistical Analysis

For data analysis, this study utilized PLS-DA, a conventional method, with the aim of achieving precise sample discrimination and screening for intergroup differential features, thereby completing multidimensional data analysis of volatile components in Cnidii Fructus samples subjected to different doses of ^60^Co irradiation [29]. To investigate the effects of varying doses of ^60^Co irradiation on differential metabolites in Cnidii Fructus, a KEGG enrichment analysis of differential metabolites was further conducted.

#### 3.2.1. Partial Least-Squares Discriminant Analysis

**Figure 3:**
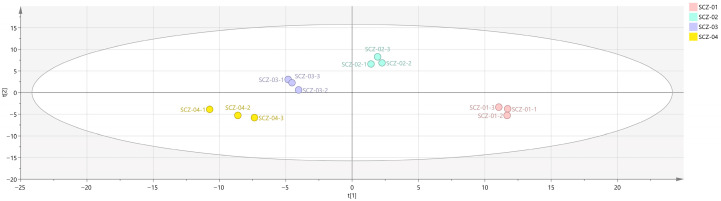
PLS-DA analysis of VOCs in four groups of Cnidii Fructus.

PLS-DA is a supervised multivariate statistical method primarily used for sample classification and discrimination studies. It combines the dimensionality reduction in partial least squares regression with the classification power of linear discriminant analysis, aiming to maximize inter-group differences and minimize intra-group variability. This approach enables effective differentiation and discrimination between different sample classes, with the characteristic feature of this analytical model being cross-validation analysis based on a specified sample size [30]. To investigate differences among the four groups of Cnidii Fructus samples subjected to different ^60^Co irradiation sterilization treatments, PLS-DA was performed using SIMCA software. The results are presented in Figure 3: The unirradiated group (SCZ-01) and all irradiated groups (SCZ-02, SCZ-03, SCZ-04) exhibited clear inter-group separation in the coordinate space, with no overlap of sample points between them. This indicates fundamental differences in the VOC profiles of Cnidii Fructus samples between irradiated and non-irradiated treatments. Clear separation trends were also observed among the irradiated groups themselves. Specifically, the low- and medium-dose groups (SCZ-02, SCZ-03) showed relatively close spatial distribution, whereas the high-dose group (SCZ-04) displayed a distinct distance from the low- and medium-dose groups (SCZ-02, SCZ-03) in the coordinate space. This indicates that the compositional characteristics of samples subjected to high-dose irradiation differed more markedly compared to those treated with low- and medium-dose irradiation.

**Figure 4:**
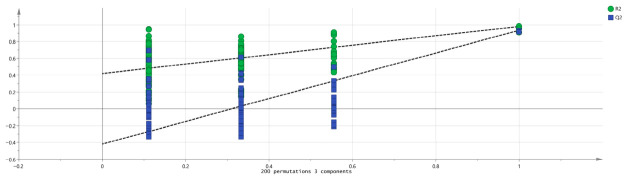
Permutation test analysis of VOCs in four groups of Cnidii Fructus.

In addition, post-processing data revealed that R^2^X = 0.399 and R^2^Y = 0.172, suggesting the model has reliable predictive capability. To check for overfitting, a 200-permutation cross-validation assessed R^2^ and Q^2^. As Figure 4 shows, R^2^ = 0.418 and Q^2^ = −0.421 were obtained. Both R^2^ and Q^2^ were lower than the original values at coordinate 1.0, and the Q^2^ regression line intersected the abscissa with a negative intercept, indicating a valid, robust, and overfitting-free statistical model.

**Figure 5:**
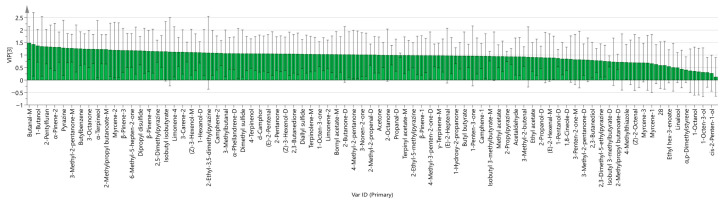
VIP values of VOCs in Cnidii Fructus subjected to different ^60^Co irradiation sterilization treatments.

The VIP value is a crucial quantitative metric for evaluating the contribution of each variable to the model’s classification and discrimination, directly reflecting the individual variables’ relative importance in distinguishing inter-group differences. By calculating the VIP values of each VOC, characteristic variables that play a central role in classifying and discriminating Cnidii Fructus samples subjected to different doses of ^60^Co irradiation can be identified, clarifying their degree of contribution to the model. Generally, a variable with a VIP value greater than 1 is considered to have a significant influence on the model’s classification and discrimination, and such components are recognized as key VOCs for differentiating between sample groups [31]. As illustrated in Figure 5 and Table 2, compounds such as Butanal-M, 1-Butanol, 2-Pentylfuran, α-Pinene-2, Pyrazine, 3-Methyl-2-pentanone-M, Butylbenzene, 3-Octanone, α-Terpineol, 2-Methylpropyl butanoate-M, Myrcene-2, β-Pinene, 6-Methyl-5-hepten-2-one, Dipropyl disulfide, 2,5-Dimethylpyrazine, Isobutyl isobutyrate, Limonene, 3-Carene, (Z)-3-Hexenol-M, 1-Hexenol-D, and 2-Ethyl-3,5-dimethylpyrazine were predominant among the VOCs in Cnidii Fructus, significantly influencing the differences in volatile components resulting from the various ^60^Co irradiation sterilization treatments.

#### 3.2.2. KEGG Enrichment Analysis of Differential Metabolites

**Figure 6:**
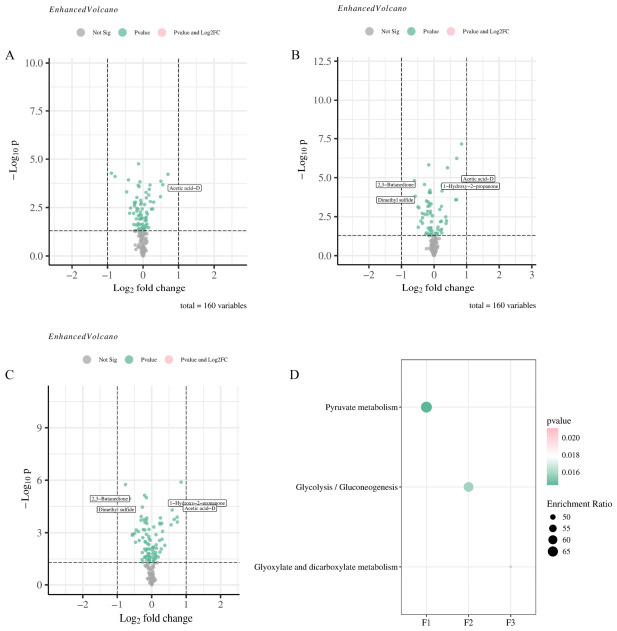
Comparison of differential metabolites and enrichment pathway analysis in Cnidii Fructus subjected to different irradiation doses. (**A**) Comparison of differential metabolites between SCZ-01 and SCZ-02; (**B**) comparison of differential metabolites between SCZ-01 and SCZ-03; (**C**) comparison of differential metabolites between SCZ-01 and SCZ-04; (**D**) overview of enriched metabolite sets (Top25). Pvalue and Log2FC are listed in text box.

To evaluate the effect of different doses of ^60^Co irradiation on the differential metabolites of Cnidii Fructus, differential metabolites were screened in this study based on the criteria of *p* < 0.05, |log_2_FC| > 1, and VIP > 1. The results are presented in Figure 6A–C. One differential metabolite (Acetic acid-D, up-regulated) was identified between SCZ-02 and SCZ-01, representing the smallest number. Four differential metabolites each were screened out between SCZ-03 and SCZ-01 and between SCZ-04 and SCZ-01 (1-Hydroxy-2-propanone and Acetic acid-D up-regulated; Dimethyl sulfide and 2,3-Butanedione down-regulated), showing a marked increase in number. Compared with the unirradiated sample, most differential metabolites in the three irradiated groups exhibited changing trends, suggesting that irradiation treatment promoted the generation and transformation of metabolites. To elucidate the potential mechanisms underlying the alterations in the metabolic profile of Cnidii Fructus induced by medium- and high-dose irradiation, a metabolic pathway enrichment analysis was performed on the four differential metabolites common to the SCZ-03 vs. SCZ-01 and SCZ-04 vs. SCZ-01 comparisons. The results (Figure 6D) indicated that these metabolites were primarily enriched in three core pathways: pyruvate metabolism (enrichment ratio: 50, *p* = 0.0145), glycolysis/gluconeogenesis (enrichment ratio: 55, *p* = 0.0158), and glyoxylate and dicarboxylate metabolism (enrichment ratio: 60, *p* = 0.0211).

## 4. Discussion

This study utilized GC-IMS technology coupled with multivariate statistical analysis to investigate the effects of varying ^60^Co irradiation doses on the VOCs of Cnidii Fructus. Compared to GC-MS, GC-IMS enables faster VOCs analysis of Cnidii Fructus with high sensitivity for bioactive components (e.g., α-Thujone), and its fingerprint visualization allows for direct comparison of volatile profiles across different irradiation doses (0–9 kGy). The fingerprint spectra generated in this study visually revealed differential distribution patterns of volatile components among the dose groups [23,24]. The unirradiated group contained multiple components like 2,3-Butanedione, while the low-dose group (3 kGy) had relatively higher levels of substances like α-Thujone and the medium-dose group (6 kGy) exhibited a higher content of compounds such as (Z)-3-Hexenol-D. The high-dose group (9 kGy) was mainly composed of 3-Methyl-2-butenal. This pattern suggests that low-dose irradiation causes minor perturbation to the original component profile of Cnidii Fructus, better preserving its initial chemical characteristics. In contrast, medium- and high-dose irradiation induce more extensive component transformation and content changes, with the high dose leading to a profound reorganization of the chemical composition.

Key differential components with VIP values above 1 were identified, including α-Thujone and 1-Octen-3-one, among others. Analysis of compound classes revealed that ketones (e.g., α-Thujone, 1-Octen-3-one) were abundant in the low-dose irradiation group, and their antimicrobial, anti-inflammatory, and neuroactive properties may confer unique pharmacodynamic characteristics to samples subjected to low-dose irradiation [32]. Alcohols (e.g., 1-Octen-3-ol) showed considerable variation in the medium- and high-dose groups. Given their known involvement in antimicrobial, anti-inflammatory, and plant defense processes [33], it is postulated that irradiation may activate stress defense mechanisms in Cnidii Fructus. Esters (e.g., Neryl acetate), which contribute to aroma [34], were highly abundant in the unirradiated group, aligning with the floral and fruity scent characteristics of the untreated sample. Terpenes (e.g., α-Pinene, β-Pinene) [35], core active components among the VOCs of Cnidii Fructus, exhibited a trend of degradation with increasing irradiation dose, explaining the observed decrease in terpene content in the high-dose group. These findings not only elucidate the specific effects of irradiation on the components of Cnidii Fructus but also provide a molecular-level basis for understanding the alterations in its pharmacological activity following irradiation. In contrast, Xiang et al. (2023) [27] primarily focused on qualitative changes in VOCs without exploring underlying metabolic mechanisms.

Differential metabolite analysis showed that the number of differential metabolites between SCZ-02 and SCZ-01 was minimal under low-dose irradiation (3 kGy), indicating that low-dose irradiation induces relatively limited perturbation to the metabolic profile. In contrast, under medium-dose (6 kGy) and high-dose (9 kGy) irradiation, the number of differential metabolites compared to the unirradiated group increased significantly, demonstrating that medium- and high-dose irradiation induce more extensive alterations in the metabolic profile.

Metabolic pathway enrichment analysis revealed that differential metabolites were primarily enriched in three core pathways: pyruvate metabolism, glycolysis/gluconeogenesis, and glyoxylate and dicarboxylate metabolism. Irradiation-induced oxidative stress activates glycolytic flux, leading to the accumulation of dihydroxyacetone phosphate, which enters the methylglyoxal pathway to generate 1-Hydroxy-2-propanone [36,37]. Simultaneously, impairment of the TCA cycle redirects pyruvate towards acetyl-CoA, which is subsequently hydrolyzed to acetic acid [37,38], resulting in the accumulation of Acetic acid-D. The redistribution of pyruvate metabolism also inhibits the acetoin pathway [39,40], leading to reduced synthesis of 2,3-Butanedione. The downregulation of Dimethyl sulfide reflects the preferential utilization of methionine for glutathione synthesis under irradiation stress to counteract oxidative damage [41,42], rather than for the production of volatile sulfur compounds. These metabolic mechanisms elucidate how irradiation alters the composition of VOCs in Cnidii Fructus by influencing core metabolic pathways.

This study found that the VOCs of Cnidii Fructus were most similar to the non-irradiated control group when the irradiation dose was 3 kGy, suggesting that its component spectrum may be closer to the characteristics of traditional high-quality medicinal materials. The identified differential metabolites and core metabolic pathways can provide a chemical reference for the quality control of irradiated Cnidii Fructus. The findings offer a theoretical basis and technical support for the rational application of ^60^Co irradiation sterilization in the processing of Chinese medicinal materials and their powders. The level of detail and specificity provided is in line with the findings of Xiang et al. (2023) [27] and offers some fresh perspectives for the field.

It is worth noting that this study only analyzed the effect of different doses of ^60^Co irradiation on the VOCs profiles of Cnidii Fructus using GC-IMS and did not detect the microbial load of the irradiated samples. Therefore, although this study found that a 3 kGy irradiation dose had minimal impact on the VOC profiles, this result indicates that the volatile chemical components of Cnidii Fructus are well retained at this dose. Subsequent microbiological testing can be conducted to evaluate the sterilization efficiency of different irradiation doses.

## 5. Conclusions

The influence of different ^60^Co doses on the VOCs of Cnidii Fructus is dose-dependent, with unique trends in compositional changes observed at low (3 kGy), medium (6 kGy), and high (9 kGy) doses. Medium- and high-dose irradiation primarily reconstructs the metabolic profile by disrupting core carbon pathways. This study identified differential metabolites and core metabolic pathways that can serve as a chemical reference for quality control of irradiated Cnidii Fructus. When the irradiation dose is 3 kGy, the VOCs profile of Cnidii Fructus is most similar to that of the non-irradiated control group, suggesting that its compositional profile may be closer to that of traditional high-quality medicinal materials. The findings provide a theoretical basis and technical support for the rational application of ^60^Co irradiation sterilization in the processing of Chinese medicinal materials and their powders.

## Figures and Tables

**Table 1:** Details of the VOCs in Cnidii Fructus with different ^60^Co irradiation intensities.

No	Compound	CAS	Formula	MW	RI	Rt/s	Dt (RIPrel)
1	Carvone	99-49-0	C_10_H_14_O	150.2	1916.8	3082.922	1.30405
2	Borneol-M	507-70-0	C_10_H_18_O	154.3	1906.1	3012.581	1.22256
	Borneol-D	507-70-0	C_10_H_18_O	154.3	1906.1	3012.581	1.88372
3	α-Terpineol	98-55-5	C_10_H_18_O	154.3	1831.4	2563.481	1.21886
4	4-Terpinenol	562-74-3	C_10_H_18_O	154.3	1700.0	1929.577	1.22000
5	Bornyl acetate-M	76-49-3	C_12_H_20_O_2_	196.3	1641.5	1700.752	1.22335
	Bornyl acetate-D	76-49-3	C_12_H_20_O_2_	196.3	1642.0	1702.364	2.18485
6	d-Camphor	464-49-3	C_10_H_16_O	152.2	1599.3	1552.499	1.34730
7	Isomenthone-M	491-07-6	C_10_H_18_O	154.3	1523.3	1317.228	1.34898
	Isomenthone-D	491-07-6	C_10_H_18_O	154.3	1523.8	1318.839	1.84481
8	Benzaldehyde-M	100-52-7	C_7_H_6_O	106.1	1556.1	1413.915	1.15802
	Benzaldehyde-D	100-52-7	C_7_H_6_O	106.1	1556.2	1414.504	1.47569
9	Acetic acid-M	64-19-7	C_2_H_4_O_2_	60.1	1503.0	1260.827	1.06086
	Acetic acid-D	64-19-7	C_2_H_4_O_2_	60.1	1503.8	1262.955	1.16933
10	Furfural	1998/1/1	C_5_H_4_O_2_	96.1	1500.1	1252.770	1.09436
11	α, p-Dimethylstyrene	1195-32-0	C_10_H_12_	132.2	1455.3	1137.341	1.17147
12	α-Thujone	546-80-5	C_10_H_16_O	152.2	1426.0	1067.617	1.37736
13	(Z)-2-Octenal	20664-46-4	C_8_H_14_O	126.2	1421.0	1056.170	1.33092
14	Nonanal-M	124-19-6	C_9_H_18_O	142.2	1411.4	1034.317	1.49192
	Nonanal-D	124-19-6	C_9_H_18_O	142.2	1412.0	1035.687	1.95161
15	2-Propylpyrazine	18138-03-9	C_7_H_10_N_2_	122.2	1434.1	1086.349	1.19005
16	(E)-3-Hexen-1-ol	928-97-2	C_6_H_12_O	100.2	1377.1	960.431	1.25816
17	1-Hexanol-M	111-27-3	C_6_H_14_O	102.2	1377.5	961.238	1.33443
	1-Hexanol-D	111-27-3	C_6_H_14_O	102.2	1377.9	962.118	1.64700
18	6-Methyl-5-hepten-2-one	110-93-0	C_8_H_14_O	126.2	1355.6	916.930	1.17848
19	cis-3-Hexenyl acetate	3681-71-8	C_8_H_14_O_2_	142.2	1328.4	864.500	1.30059
20	1-Hydroxy-2-propanone	116-09-6	C_3_H_6_O_2_	74.1	1320.8	850.469	1.07696
21	Octanal-M	124-13-0	C_8_H_16_O	128.2	1307.8	826.838	1.41535
	Octanal-D	124-13-0	C_8_H_16_O	128.2	1307.6	826.571	1.81937
22	Terpinolene-M	586-62-9	C_10_H_16_	136.2	1298.1	809.854	1.21820
	Terpinolene-D	586-62-9	C_10_H_16_	136.2	1297.7	809.115	1.30059
23	Hexyl acetate	142-92-7	C_8_H_16_O_2_	144.2	1291.1	798.038	1.38593
24	β-Ocimene	13877-91-3	C_10_H_16_	136.2	1272.4	768.248	1.21431
25	1-Pentanol-M	71-41-0	C_5_H_12_O	88.1	1272.1	767.659	1.25450
	1-Pentanol-D	71-41-0	C_5_H_12_O	88.1	1272.6	768.513	1.51872
26	γ-Terpinene-M	99-85-4	C_10_H_16_	136.2	1261.8	751.756	1.21144
	γ-Terpinene-D	99-85-4	C_10_H_16_	136.2	1261.8	751.756	1.70235
27	(E)-2-Hexenal-M	6728-26-3	C_6_H_10_O	98.1	1241.4	721.127	1.17842
	(E)-2-Hexenal-D	6728-26-3	C_6_H_10_O	98.1	1241.5	721.281	1.51373
28	1,8-Cineole-M	470-82-6	C_10_H_18_O	154.3	1224.2	696.389	1.30187
	1,8-Cineole-D	470-82-6	C_10_H_18_O	154.3	1223.8	695.800	1.72532
29	Limonene	138-86-3	C_10_H_16_	136.2	1216.3	685.198	1.21574
30	Heptanal	111-71-7	C_7_H_14_O	114.2	1206.9	672.239	1.35067
31	α-Terpinene-M	99-86-5	C_10_H_16_	136.2	1195.2	656.336	1.21431
	α-Terpinene-D	99-86-5	C_10_H_16_	136.2	1195.6	656.925	1.72101
32	Myrcene	123-35-3	C_10_H_16_	136.2	1183.8	633.953	1.21431
33	p-Xylene	106-42-3	C_8_H_10_	106.2	1154.4	573.285	1.07938
34	3-Carene	13466-78-9	C_10_H_16_	136.2	1145.0	555.026	1.21431
35	β-Pinene	127-91-3	C_10_H_16_	136.2	1131.3	529.698	1.21431
36	Isoamyl acetate	123-92-2	C_7_H_14_O_2_	130.2	1149.9	564.450	1.30474
37	Hexanal-M	66-25-1	C_6_H_12_O	100.2	1112.5	496.683	1.27735
	Hexanal-D	66-25-1	C_6_H_12_O	100.2	1112.8	497.151	1.55548
38	Camphene	79-92-5	C_10_H_16_	136.2	1096.2	469.780	1.20959
39	α-Pinene	80-56-8	C_10_H_16_	136.2	1049.8	411.008	1.21650
40	1-Butanol	71-36-3	C_4_H_10_O	74.1	1167.6	599.741	1.18469
41	3-Methyl-2-pentanone-M	565-61-7	C_6_H_12_O	100.2	1039.6	399.015	1.17635
	3-Methyl-2-pentanone-D	565-61-7	C_6_H_12_O	100.2	1039.8	399.273	1.47649
42	1-Penten-3-one	1629-58-9	C_5_H_8_O	84.1	1026.1	383.827	1.09059
43	Ethanol-M	64-17-5	C_2_H_6_O	46.1	960.9	325.908	1.04112
	Ethanol-D	64-17-5	C_2_H_6_O	46.1	961.6	326.423	1.12577
44	2-Propanol-M	67-63-0	C_3_H_8_O	60.1	949.9	318.186	1.09389
	2-Propanol-D	67-63-0	C_3_H_8_O	60.1	950.6	318.701	1.22032
45	3-Methylbutanal	590-86-3	C_5_H_10_O	86.1	945.4	315.097	1.40173
46	2-Butanone-M	78-93-3	C_4_H_8_O	72.1	931.7	305.830	1.06311
	2-Butanone-D	78-93-3	C_4_H_8_O	72.1	931.7	305.830	1.24671
47	Ethyl acetate	141-78-6	C_4_H_8_O_2_	88.1	914.3	294.421	1.09618
48	2-Methyl-2-propenal-M	78-85-3	C_4_H_6_O	70.1	912.8	293.421	1.04990
	2-Methyl-2-propenal-D	78-85-3	C_4_H_6_O	70.1	912.8	293.421	1.22164
49	Butanal-M	123-72-8	C_4_H_8_O	72.1	907.6	290.135	1.13731
	Butanal-D	123-72-8	C_4_H_8_O	72.1	907.8	290.259	1.28262
50	2-Propenal	107-02-8	C_3_H_4_O	56.1	881.1	273.848	1.06224
51	Methyl acetate	79-20-9	C_3_H_6_O_2_	74.1	864.3	263.991	1.19285
52	Acetone	67-64-1	C_3_H_6_O	58.1	856.6	259.562	1.11572
53	2-Methylpropanal	78-84-2	C_4_H_8_O	72.1	850.5	256.133	1.27923
54	Propanal-M	123-38-6	C_3_H_6_O	58.1	836.8	248.561	1.07047
	Propanal-D	123-38-6	C_3_H_6_O	58.1	836.8	248.561	1.14760
55	Dimethyl sulfide	75-18-3	C_2_H_6_S	62.1	811.9	235.418	0.95426
56	Acetaldehyde	75-07-0	C_2_H_4_O	44.1	786.2	222.560	1.03345
57	Neryl acetate	141-12-8	C_12_H_20_O_2_	196.3	1866.0	2762.208	1.21051
58	Terpinyl acetate-M	80-26-2	C_12_H_20_O_2_	196.3	1775.1	2269.796	1.21051
	Terpinyl acetate-D	80-26-2	C_12_H_20_O_2_	196.3	1775.7	2272.429	1.71696
59	2,3-Butanediol	513-85-9	C_4_H_10_O_2_	90.1	1677.0	1836.231	1.35373
60	1-Octanol	111-87-5	C_8_H_18_O	130.2	1666.1	1793.338	1.47730
61	Linalool	78-70-6	C_10_H_18_O	154.3	1595.5	1539.598	1.21306
62	(E)-2-Nonenal	18829-56-6	C_9_H_16_O	140.2	1581.5	1493.929	1.38556
63	3-Nonen-2-one	14309-57-0	C_9_H_16_O	140.2	1563.7	1437.339	1.35758
64	2,3-Diethyl-6-methylpyrazine	18138-04-0	C_9_H_14_N_2_	150.2	1507.6	1273.476	1.30270
65	1-Octen-3-ol	3391-86-4	C_8_H_16_O	128.2	1493.0	1233.819	1.16795
66	2-Ethyl-3,5-dimethylpyrazine	13925-07-0	C_8_H_12_N_2_	136.2	1470.0	1173.927	1.19958
	2,6-Dimethyl-3-ethylpyrazine	13925-07-0	C_8_H_12_N_2_	136.2	1497.5	1245.959	1.21745
67	(Z)-3-Hexenyl isovalerate	35154-45-1	C_11_H_20_O_2_	184.3	1505.3	1267.002	1.46771
68	Linalool oxide	1365-19-1	C_10_H_18_O_2_	170.3	1481.3	1203.064	1.25320
69	2,3-Dimethyl-5-ethylpyrazine	15707-34-3	C_8_H_12_N_2_	136.2	1471.9	1178.783	1.24633
70	(E)-2-Octenal	2548-87-0	C_8_H_14_O	126.2	1453.4	1132.651	1.33983
71	5-Methyl-2(3H)-furanone	591-12-8	C_5_H_6_O_2_	98.1	1420.4	1054.729	1.12347
72	(Z)-3-Hexenol-M	928-96-1	C_6_H_12_O	100.2	1410.4	1032.225	1.24449
	(Z)-3-Hexenol-D	928-96-1	C_6_H_12_O	100.2	1410.0	1031.360	1.51238
73	2-Ethyl-5-methylpyrazine	13360-64-0	C_7_H_10_N_2_	122.2	1404.2	1018.377	1.19282
74	cis-2-Penten-1-ol	1576-95-0	C_5_H_10_O	86.1	1344.7	895.472	0.94941
75	Hexyl isobutyrate	2349/7/7	C_10_H_20_O_2_	172.3	1361.2	928.087	1.47664
76	(E)-2-Heptenal	18829-55-5	C_7_H_12_O	112.2	1337.5	881.801	1.25463
77	2,6-Dimethylpyrazine	108-50-9	C_6_H_8_N_2_	108.1	1331.7	870.747	1.13218
78	2,5-Dimethylpyrazine	123-32-0	C_6_H_8_N_2_	108.1	1318.1	845.515	1.11026
79	1,2,3-Trimethylbenzene	526-73-8	C_9_H_12_	120.2	1323.3	855.037	1.13972
80	Ethyl hex-3-enoate	2396-83-0	C_8_H_14_O_2_	142.2	1325.5	859.192	1.25107
81	1-Octen-3-one	4312-99-6	C_8_H_14_O	126.2	1320.0	849.042	1.28014
82	Butylbenzene	104-51-8	C_10_H_14_	134.2	1318.8	846.867	1.22142
83	3-Methyl-2-butenyl acetate	1191-16-8	C_7_H_12_O_2_	128.2	1276.8	775.107	0.94593
84	2-Octanone	111-13-7	C_8_H_16_O	128.2	1304.8	821.497	1.33780
85	2-Methyltetrahydrofuran-3-one	3188-00-9	C_5_H_8_O_2_	100.1	1291.0	797.849	1.44419
86	4-Methylthiazole	693-95-8	C_4_H_5_NS	99.2	1283.0	785.015	1.34405
87	Styrene	100-42-5	C_8_H_8_	104.2	1276.5	774.625	1.05410
88	3-Octanone	106-68-3	C_8_H_16_O	128.2	1274.6	771.569	1.30562
89	Butyl 2-methylbutanoate	15706-73-7	C_9_H_18_O_2_	158.2	1248.8	732.081	1.37962
90	(Z)-4-Heptenal	6728-31-0	C_7_H_12_O	112.2	1236.4	713.918	1.14788
91	3-Methyl-2-butenal	107-86-8	C_5_H_8_O	84.1	1223.1	694.772	1.09102
92	3-Methyl-1-butanol	123-51-3	C_5_H_12_O	88.1	1232.0	707.536	1.25088
93	Pyrazine	290-37-9	C_4_H_4_N_2_	80.1	1204.0	668.262	1.04596
94	2-Heptanone	110-43-0	C_7_H_14_O	114.2	1201.3	664.574	1.26192
95	Butyl butyrate	109-21-7	C_8_H_16_O_2_	144.2	1211.7	678.891	1.82706
96	Isobutyl 3-methylbutyrate-M	589-59-3	C_9_H_18_O_2_	158.2	1192.1	652.302	1.36947
	Isobutyl 3-methylbutyrate-D	589-59-3	C_9_H_18_O_2_	158.2	1192.1	652.302	1.88794
97	2-Methylpropyl butanoate-M	539-90-2	C_8_H_16_O_2_	144.2	1166.5	597.488	1.35020
	2-Methylpropyl butanoate-D	539-90-2	C_8_H_16_O_2_	144.2	1166.7	597.897	1.84330
98	o-Xylene	95-47-6	C_8_H_10_	106.2	1179.0	623.668	1.06813
99	α-Phellandrene-M	99-83-2	C_10_H_16_	136.2	1173.6	612.214	1.21525
	α-Phellandrene-D	99-83-2	C_10_H_16_	136.2	1173.8	612.623	1.68603
100	Diallyl sulfide	592-88-1	C_6_H_10_S	114.2	1154.7	573.837	1.33320
101	3-Penten-2-one-M	625-33-2	C_5_H_8_O	84.1	1125.2	518.684	1.09021
	3-Penten-2-one-D	625-33-2	C_5_H_8_O	84.1	1125.4	519.106	1.34559
102	Isobutyl isobutyrate	97-85-8	C_8_H_16_O_2_	144.2	1117.0	504.328	1.79920
103	2,3-Pentanedione	600-14-6	C_5_H_8_O_2_	100.1	1065.5	430.016	1.21148
104	Pentanal	110-62-3	C_5_H_10_O	86.1	1015.0	371.749	1.42022
105	2-Pentanone	107-87-9	C_5_H_10_O	86.1	1011.9	368.371	1.36774
106	2,3-Butanedione	431-03-8	C_4_H_6_O_2_	86.1	1009.5	365.838	1.18233
107	4-Methyl-2-pentanone	108-10-1	C_6_H_12_O	100.2	1029.7	387.793	1.47735
108	2-Acetylfuran	1192-62-7	C_6_H_6_O_2_	110.1	1542.1	1372.061	1.11274
109	2-Nonanone	821-55-6	C_9_H_18_O	142.2	1407.5	1025.637	1.41337
110	Dipropyl disulfide	629-19-6	C_6_H_14_S_2_	150.3	1390.0	987.559	1.24589
111	2-Pentylfuran	3777-69-3	C_9_H_14_O	138.2	1252.8	738.145	1.25151
112	4-Methyl-3-penten-2-one-M	141-79-7	C_6_H_10_O	98.1	1160.1	584.582	1.12450
	4-Methyl-3-penten-2-one-D	141-79-7	C_6_H_10_O	98.1	1160.2	584.795	1.44714
113	Pentyl acetate	628-63-7	C_7_H_14_O_2_	130.2	1162.7	589.783	1.30947
114	(E)-2-Pentenal	1576-87-0	C_5_H_8_O	84.1	1157.4	579.220	1.35916
115	2-Methyl-1-propanol	78-83-1	C_4_H_10_O	74.1	1118.4	506.748	1.17077

Note: The substance suffixes M and D represent monomers and dimers of the same substance, respectively. RIPrel represents the normalized processing of reactive ion peaks.

**Table 2:** Details of the VOCs with a VIP value greater than 1 in Cnidii Fructus under different ^60^Co irradiation intensities.

No	Compound	VIP
1	Butanal-M	1.48739
2	α-Thujone	1.4336
3	1-Butanol	1.37854
4	Hexanal-M	1.35517
5	2-Pentylfuran	1.34109
6	α-Phellandrene-M	1.33123
7	α-Pinene-2	1.32872
8	2,3-Pentanedione	1.32122
9	Pyrazine	1.29628
10	3-Methyl-2-pentanone-M	1.28259
11	Ethanol-D	1.27871
12	Butylbenzene	1.25354
13	Isomenthone-M	1.24575
14	α-Terpineol	1.24197
15	Ethanol-M	1.24131
16	3-Octanone	1.23559
17	Isomenthone-D	1.2345
18	2-Methylpropyl butanoate-M	1.23268
19	α-Pinene-3	1.20798
20	Myrcene-2	1.20646
21	Isoamyl acetate	1.20401
22	β-Pinene-3	1.19601
23	1-Hexanol-M	1.18891
24	3-Carene-3	1.18791
25	6-Methyl-5-hepten-2-one	1.18495
26	Linalool oxide	1.17649
27	Dipropyl disulfide	1.17083
28	β-Pinene-4	1.15893
29	Benzaldehyde-D	1.15726
30	2,5-Dimethylpyrazine	1.14909
31	Benzaldehyde-M	1.14513
32	Isobutyl isobutyrate	1.14327
33	Camphene-3	1.13633
34	Limonene-4	1.12932
35	Hexyl isobutyrate	1.12532
36	3-Carene-2	1.12328
37	2-Propanol-M	1.1146
38	Myrcene-4	1.11333
39	(Z)-3-Hexenol-M	1.10833
40	1-Hexanol-D	1.10287
41	2-Ethyl-3,5-dimethylpyrazine	1.09706
42	Limonene-1	1.09644
43	Camphene-2	1.08682
44	2-Propenal	1.0844
45	2-Heptanone	1.08097
46	1,2,3-Trimethylbenzene	1.07268
47	α-Phellandrene-D	1.06808
48	2-Nonanone	1.06765
49	3-Methylbutanal	1.06267
50	(E)-2-Pentenal	1.06128
51	2,3-Diethyl-6-methylpyrazine	1.05916
52	d-Camphor	1.05855
53	4-Terpinenol	1.05781
54	2-Methylpropanal	1.05723
55	Dimethyl sulfide	1.0562
56	γ-Terpinene-D	1.05413
57	3-Carene-1	1.05395
58	Terpinolene-D	1.05321
59	2-Pentanone	1.05199
60	(Z)-3-Hexenol-D	1.0471
61	Propanal-M	1.0455
62	2,3-Butanedione	1.04374
63	Nonanal-D	1.04212
64	Diallyl sulfide	1.03801
65	Hexanal-D	1.03602
66	Acetic acid-M	1.03381
67	Terpinolene-M	1.03363
68	1-Octen-3-one	1.03262
69	2-Butanone-D	1.02998
70	α-Terpinene-D	1.02992
71	Limonene-2	1.02841
72	4-Methyl-2-pentanone	1.02784
73	3-Methyl-1-butanol	1.02536
74	Bornyl acetate-M	1.02272
75	Carvone	1.02232
76	Acetic acid-D	1.01986
77	2-Methyl-2-propenal-D	1.01517
78	3-Nonen-2-one	1.01429
79	5-Methyl-2(3H)-furanone	1.01342
80	Butanal-D	1.01037
81	Furfural	1.00798

## Data Availability

The original contributions presented in the study are included in the article, and further inquiries can be directed to the corresponding author.
